# A trio of biological rhythms and their relevance in rhythmic mechanical stimulation of cell cultures

**DOI:** 10.3389/fpsyg.2022.867191

**Published:** 2022-07-29

**Authors:** Dongho Kwak, Petter Angell Olsen, Anne Danielsen, Alexander Refsum Jensenius

**Affiliations:** ^1^Department of Musicology, RITMO Centre for Interdisciplinary Studies in Rhythm, Time and Motion, University of Oslo, Oslo, Norway; ^2^Hybrid Technology Hub-Centre for Organ on a Chip-Technology, Institute of Basic Medical Sciences, University of Oslo, Oslo, Norway; ^3^Unit for Cell Signaling, Department of Immunology and Transfusion Medicine, Oslo University Hospital, Oslo, Norway

**Keywords:** biological rhythms, cellular rhythms, rhythmic mechanical stimulation, cell cultures, tissue engineering, regenerative medicine

## Abstract

The primary aim of this article is to provide a biological rhythm model based on previous theoretical and experimental findings to promote more comprehensive studies of rhythmic mechanical stimulation of cell cultures, which relates to tissue engineering and regenerative medicine fields. Through an interdisciplinary approach where different standpoints from biology and musicology are combined, we explore some of the core rhythmic features of biological and cellular rhythmic processes and present them as a trio model that aims to afford a basic but fundamental understanding of the connections between various biological rhythms. It is vital to highlight such links since rhythmic mechanical stimulation and its effect on cell cultures are vastly underexplored even though the cellular response to mechanical stimuli (mechanotransduction) has been studied widely and relevant experimental evidence suggests mechanotransduction processes are rhythmic.

## Introduction

*Rhythm* is one of the most basic and important elements in music. It usually has a repetitive structure typical of rhythmic signals but is also characterized by small and large deviations from that structure. Accordingly, we think of rhythms as ordered patterns in time. The importance of rhythm in music is comparable with that of rhythm in biological systems: rhythm in music is not trivial but one of the essential devices for musical expressions and an element that makes music “alive,” and likewise, rhythm in biological systems is not only an observable phenomenon but necessary for sustaining life. As fundamental biological phenomena (Haken and Koepchen, 1991), rhythmic biological processes are related to the tendency to stay in balance between chaos and order (i.e., homeostasis; Crutchfield, 2003; Gnocchi and Bruscalupi, 2017). We contend that this rhythmic “balancing act” of homeostasis is one of the key biological elements that is insufficiently accentuated and overlooked in the research area of mechanical stimulation of cell cultures in relation to tissue engineering and regenerative medicine fields, in which providing and mimicking a dynamic *in vivo* environment for *in vitro* cell culture models is an important question.

One of the major developments in tissue engineering has been related to micro-scale structural engineering. For example, spatial variations and their effects on cell cultures have been studied extensively by using various types of technologies such as 3D culturing systems, bioprinting, and organ-on-chip designs (Kim and Hayward, 2012; Lee and Cho, 2016; Jensen and Teng, 2020; Low et al., 2021). The main advantages provided by the intricate structural designs include growing cells in various patterns and shapes and on different material stiffness (e.g., gel or PDMS), which create specific types and varying degrees of mechanical restrictions and forces on the cell cultures. As a result of such improvements, along with recent developments in stem cell technologies, it is now possible to generate organoids and mini tissues that represent the functional characteristics of the organ from which the stem cells were derived (Kratochvil et al., 2019). Optimization of the structural environment of cell culture systems is actively being pursued to advance the development of personalized medicine and drug screening (Kim et al., 2020). However, as biological processes occur spatiotemporally (Grace and Hütt, 2015), what should be as critical as optimal mechanical stimulation by structural cues (i.e., ordered patterns in space) is the optimization of temporal patterns of the mechanical stimulation (i.e., ordered patterns in time). Rhythmic stimulations have been explored, but only in a small number of areas, such as microfluidic systems used on blood vessel cells (endothelial cells; Novo et al., 2016; Yeom et al., 2017; Ortseifen et al., 2020), electrical stimulations used on cardiac cells (cardiomyocytes; Laasmaa et al., 2019), and application of cyclic tensile strain to mimic respiratory motions in lung-on-chip platforms (Huh et al., 2010).

In this article, firstly, we briefly present an overview of biological rhythms in different temporal scales. Secondly, we present a trio biological rhythm model in terms of central rhythm, internal/external rhythm, and reflex/consequential rhythm and discuss how these rhythms are interconnected to regulate homeostasis in a biological system. Thirdly, we explore selected biological and cellular rhythms with critical functions that demonstrate the trio rhythm model in human body organ systems, such as the cardiovascular system (specifically rhythms in relation to blood pressure, blood vessels, and smooth muscle cells) and the digestive system (pancreas, β-cells, and insulin secretion). Lastly, we discuss the potential relevance of the presented trio rhythm model and cellular rhythm examples in the context of rhythmic mechanical stimulation—using various types of experimental apparatuses that can generate controlled mechanical/physical forces such as compression, tension, and shear force directly on the cell cultures—of cell cultures. This article aims to shed light on the rhythmic mechanical stimulation of cell cultures as an area that deserves more consideration in terms of the design of cell culture systems and other cellular experiments in general, but not to provide exhaustive descriptions of biological rhythms or to investigate the origin of biological rhythms, which has been previously discussed in-depth in Haken and Koepchen (1991) and Glass (2001).

## Rhythms in different temporal scales

**Figure 1:**
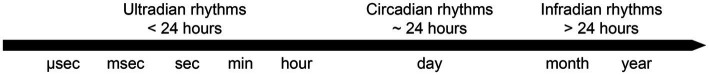
A basic illustration of three levels of biological rhythm cycles in different time scales.

The biological rhythms can be subdivided largely into three levels in terms of temporal scales. Firstly, as shown in Figure 1, ultradian rhythms refer to recurring cycles that are completed more than once per day. For example, molecular and cellular rhythms can occur within seconds to several hours (Goldbeter et al., 2012). Larger structures like cardiovascular and respiratory systems also have rhythms functioning within this scale (Haken and Koepchen, 1991). Secondly, circadian rhythms, which are studied more broadly than the other two rhythms, are recurring cycles completed daily. These rhythms are mainly activated by light and dark patterns. A generally known example would be sleep and wake cycles (Van Someren, 2000; Tähkämö et al., 2019). Thirdly, infradian rhythms refer to recurring cycles which can last longer than a day. The cycles in this time scale can last for months and years. These rhythms include menstrual cycles, human life cycles, and generations of life.

Even though these three rhythm levels are presented as independent levels, there are possible interactions between them (Laje et al., 2018). For example, ultradian rhythms may be harmonics—integer multiples of the fundamental frequency—of 24-h circadian rhythms (Zhu et al., 2018). In some cases, interactions may result in entrainment—the interaction of independent rhythmic systems—between rhythms at different time scales (Kuhlman et al., 2018). This pertains to cellular rhythms *in vivo* that involve changes in melatonin levels and sleep–wake cycles (Gnocchi and Bruscalupi, 2017). For example, one of the main factors that regulate insulin secretion rhythms in β-cells (ultradian and circadian), apart from other factors such as rhythmic inter- and intracellular calcium ion (Ca^2+^) levels (Daraio et al., 2017), is the circadian melatonin rhythm in the body (Perelis et al., 2016). We will discuss some of these fundamental rhythms and their interrelationship in the following section.

## Homeostatic rhythms

### A trio of rhythms

**Figure 2:**
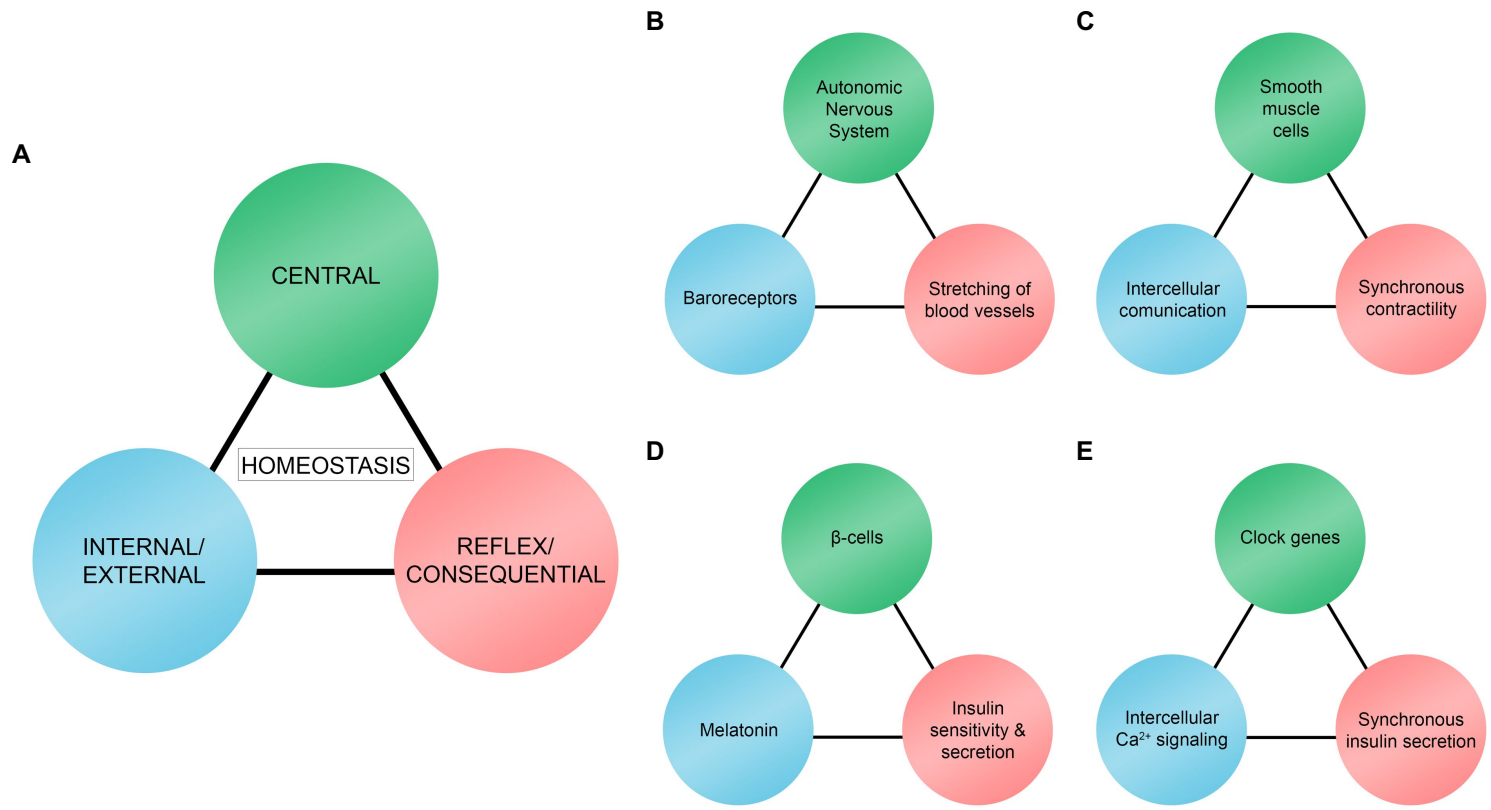
Trio model of central rhythms, internal/external rhythms, and reflex/consequential rhythms. **(A)** Homeostasis results from interactions between the trio rhythms. **(B)** Interaction between the autonomic nervous system, baroreceptors, and mechanical stretching of blood vessels to regulate and maintain blood pressure. **(C)** Interaction between smooth muscle cells, intercellular communication, and synchronous contractility of the cells to regulate and maintain rhythmic contractility of smooth muscle cells. **(D)** Interaction between β-cells, melatonin, and insulin sensitivity and secretion as one of the mechanisms to regulate and maintain glucose levels. **(E)** Interaction between clock genes, intracellular Ca^2+^ signaling, and synchronous insulin secretion as one of the mechanisms to regulate and maintain glucose levels.

We here illustrate biological rhythms as a trio model (Figure 2A). The three rhythms act as stimuli hierarchically, sequentially, and reciprocally depending on the context. The main objective of the trio is to maintain and regulate homeostasis in a given system. The trio model resembles the homeostatic model suggested by Modell et al. (2015). According to their model, there must be (a) sensors or receptors, (b) a control center for integrating and processing received information, and (c) effectors in homeostatic systems. These are comparable to what we will refer to as central rhythms (control center), internal/external rhythms (sensors or receptors), and reflex/consequential rhythms (effectors). Although our model is similar to the one presented by Modell et al. (2015), there are some differences. In our trio model, internal/external rhythms are not always sensors or receptors. They are more comprehensive and include rhythmic phenomena (e.g., intercellular communication, Ca^2+^ signaling, and melatonin level) that have an essential role in maintaining homeostasis processes in different temporal scales. Moreover, reflex/consequential rhythms pertain more to rhythmic biological responses or phenomena that are different from effectors. Effectors are typically locations or targets that the control center sends signals to, including cells, tissues, and organs (Modell et al., 2015).

In our model, central rhythms are often coming from a specific central location [e.g., Autonomic Nervous System (ANS), smooth muscle cells, and pancreatic β-cells in our examples, which are discussed in the next section] where information is gathered, integrated, and processed. Some typical examples of central rhythms can be the brain, the nervous system, and the nucleus of the cells. Central rhythms are also rhythmic biological phenomena that play a central role in the trio, such as the clock genes regulating synchronous insulin secretion in β-cells.

Central rhythms receive information from and work synchronously with internal/external rhythms. Internal rhythms are endogenous (located or generated within the location of the central rhythms), such as intercellular communication within the group of smooth muscle cells (Figure 2C) and intracellular Ca^2+^ signaling in β-cells (Figure 2E). External rhythms are exogenous (located or generated outside the central rhythms), such as baroreceptors (Figure 2B) and melatonin (Figure 2D) that are located and generated, respectively, outside the location of the central rhythms. Internal/external rhythms signal the central rhythms of the changes occurring in their immediate environment. Internal/external rhythms are often used as experimental variables that are manipulatable such as levels of melatonin (Pourhanifeh et al., 2020) and Ca^2+^ (Cavieres-Lepe and Ewer, 2021), and electrically activated baroreceptors (Tohyama et al., 2020).

As a result of interactions between central and internal/external rhythms, reflex/consequential rhythms take place. They are either negative or positive deterministic results of the interaction between the first two rhythms. For example, these rhythms are shown through stretching of blood vessels to maintain blood pressure homeostasis (Figure 2B; reflex), enhanced or activation of synchronous smooth muscle cells contractility (Figure 2C; consequential), and increased insulin sensitivity and secretion in β-cells (Figures 2D,E; consequential and reflex respectively). However, they can also interact with other rhythms to create a feedback loop. For instance, the stretching rate of the blood vessel walls keeps baroreceptors updated (Figure 2B).

The three rhythms have their unique rhythmicity and they constantly interact. This is another point that we agree with the model given by Modell et al. (2015), where the signal flow is perpetual. The interactions result in rhythms that are balanced (not chaotic but not rigidly regular) observed as homeostasis in healthy biological systems.

What is interesting is that the three homeostatic rhythms may be interconnected in a broader network of trio rhythm models at different temporal scales. For example, Figures 2B,C are linked in a way that the smooth muscle cells (central rhythms in Figure 2C) are located within blood vessels (reflex/consequential rhythms in Figure 2B) as a sub-rhythmic component and Figures 2D,E are linked in a way that the clock genes (central rhythms in Figure 2E) are located within β-cells (central rhythms in Figure 2D).

In the following section, we look into selected examples of vital rhythmic phenomena in human organ systems. These examples show that taking rhythms and their interplay into consideration provides a holistic perspective of the biological rhythmic system. All three rhythms in the trio must be continuously and simultaneously active to maintain balance, and each set might be coupled to another within and across different spatial and temporal scales. Thus, the pattern may exist regardless of the size of the system, such as the human body, organ systems, or cells.

### Cardiovascular rhythms

Heart rate is one of the rhythmic biological phenomena in the human body that are noticeable and crucial for sustaining life (Thaulow and Erikssen, 1991). In the regulation process of heart rates and blood pressure, baroreceptors are one of the components of the cardiovascular system that play an important role. Baroreceptors are sensors that detect mechanical properties of blood vessels that can be divided into two types: high-pressure arterial and low-pressure volume receptors (Armstrong et al., 2021). Both subtypes are stimulated by the stretching of blood vessel walls and transmit nerve impulses rhythmically to the ANS (Suarez-Roca et al., 2019; Armstrong et al., 2021). As a result of the rhythmic systole and diastole of the heart, blood vessels rhythmically and passively stretch to accommodate the pulsatile blood flow (Camasão and Mantovani, 2021). For example, when the stretching rate of the blood vessels is increased, the impulse firing rate of the baroreceptors will also be higher. Consequently, stimulation of the nucleus tractus solitarius region in the brain stem will lead to increased inhibition of cardiac output (i.e., decreased blood volume and pressure). Thus, a negative feedback loop is created that lowers the stretching rate of the blood vessel walls (Armstrong et al., 2021). This perpetual rhythmic process is called the baroreceptor reflex (Armstrong et al., 2021). Blood pressure is maintained and regulated (homeostasis; Figure 2) through interaction between the ANS (central rhythm), baroreceptors (external rhythm), and blood vessels (reflex rhythm). This interaction is one of the main components that make up the rhythmicity of the cardiovascular system. The rhythmic balance in this particular system is vital. Baroreceptors may influence blood pressure variability, and their decreased function is related to severe medical conditions such as hypertension (Tohyama et al., 2020; Ziegler, 2021).

In this model, mechanical changes of blood vessels (reflex rhythm) are due to continuous dynamic changes in blood flow depending on the blood pressure and volume that generates distension pressure on the vessel walls (Anwar et al., 2012). Apart from the baroreceptor reflex, evidence suggests that the communication between single cells is also important for synchronous rhythmic contractility of the blood vessels.

Blood vessels are a multilayered structure consisting of inner (tunica intima), middle (tunica media), and outer (tunica adventitia) cell layers (Tucker et al., 2021). Smooth muscle cells are found in the middle layer and contribute to the strength and contractility of blood vessels (Anwar et al., 2012). Interestingly, blood vessels display rhythmic activities when nerve signaling has been blocked (denervation; Siegel et al., 1991). It has been shown that this autorhythmic behavior of smooth muscle cells is regulated by intercellular communication (Koepchen, 1991; Siegel et al., 1991). This is achieved through gap junctions which are channels that permit the transfer of ions and small molecules between cells (Ross and Pawlina, 2003). Through the gap junctions, the levels of Ca^2+^ are synchronized between cells, thus resulting in autorhythmic activities (Slovut, 2004). When the gap junctions are chemically inhibited, the rhythmic activities decrease substantially (Slovut, 2004).

Taken together, a rhythmic phenomenon arises from the interaction between smooth muscle cells (central rhythm), intercellular communication (internal rhythm), and synchronization (consequential rhythm) that results in a continuation of rhythmic activities and possibly contributes to the entire cardiovascular rhythms (homeostasis; Figure 2C).

### Pancreatic rhythms

The pancreas is part of the digestive organ system in the human body formed around weeks 4 and 5 of gestation (Pandol, 2010), and it consists of glands that can be largely divided into two components: exocrine and endocrine glands (Netter, 2011). Although the pancreas has been studied for many years*—*possibly since ancient times (Ceranowicz et al., 2015)*—*rhythmic activities, such as more ribosome synthesis during the day in the exocrine part of the organ, were observed and reported only a few decades ago (Volkl and Poort, 1983). Subsequently, more evidence has been accumulating to support that the pancreas is a rhythmic system. In particular, there has been growing interest in understanding more about possible correlations between circadian rhythms and core activities in the endocrine of the pancreas (e.g., insulin production and secretion), which are related to diseases such as diabetes (Marcheva et al., 2010; Sadacca et al., 2011; Vieira et al., 2013, 2014; García-Costela et al., 2020; Seshadri and Doucette, 2021).

In the pancreas, the exocrine glands help to break down nutrients by producing pancreatic enzymes, whereas the endocrine glands produce hormones, which enter directly into the bloodstream, including glucagon and insulin, to regulate the blood sugar level (Edlund, 2002). In endocrine glands, specialized groups of cells are found. These clusters, also known as the islets of Langerhans, mainly consist of four different cell types: α-, β-, δ-, and pancreatic polypeptide (PP) cells (Zhong and Jiang, 2019). Among these cells, β-cells take up the most mass of an islet (60–80%; Edlund, 2002), and they are responsible for controlling blood glucose levels by secreting hormones (i.e., insulin) in the bloodstream. Although the β-cells start to form in the early gestation stages, glucose-stimulated insulin secretion is insufficient in β-cells in neonates (Seshadri and Doucette, 2021). β-cells continue to develop during the perinatal period and show rhythmic activities only after birth.

Among various intra- and extracellular factors that are involved in the rhythmicity of β-cells (Heart and Smith, 2007; Perelis et al., 2016), rhythmic stimulation and entrainment to fasting-feeding cycles and the activation of specific circadian clock genes (*ARNTL*, *PER*, and *CRY*) may be critical factors for the postnatal maturation of β-cells (Alvarez-Dominguez et al., 2020; Seshadri and Doucette, 2021). It has been shown that the deletion of *ARNTL* (also known as *BMAL1*) inhibited the complete maturation of β-cells in rodent models (Rakshit et al., 2018). Moreover, inhibiting circadian clock genes reduced glucose-stimulated insulin secretion in β-cells even in fully matured isolated islets from both rodent and human models (Perelis et al., 2016; Saini et al., 2016). Therefore, internal and external rhythmic stimulation and entrainment of β-cells are essential for insulin secretion both in immature (during the perinatal period) and mature cells.

As an external rhythmic stimulation, the circadian rhythmic variation in melatonin protein level plays a crucial role in regulating insulin sensitivity and secretion by β-cells. Melatonin is also referred to as *Zeitgeber*, the German word for “time giver” (Pandi-Perumal et al., 2006). It is produced and secreted predominantly from a small endocrine gland in the brain called the pineal gland (Pandi-Perumal et al., 2006). However, other parts of the human body, such as the retina and skin, can also produce melatonin (Srinivasan et al., 2009). As accumulating evidence shows that circadian rhythm is an important factor in type 2 diabetes, although further investigation is necessary, it has been hypothesized that melatonin may have a therapeutic property in treating type 2 diabetes (Sharma et al., 2015). This is in line with the results from separate experimental studies. For example, treating isolated islets from rodents with melatonin overnight to mimic an *in vivo* environment promoted subsequent insulin sensitivity and secretion the following morning (Kemp et al., 2002). In a separate experimental study, a similar relationship between insulin secretion and melatonin in human type 2 diabetic patients was found: patients with decreased insulin secretion and glucose tolerance had reduced melatonin productions (Pourhanifeh et al., 2020). In this specific context, homeostatic interaction arises between β-cells (central rhythm), melatonin (external rhythm), and insulin sensitivity and secretion (reflex rhythm), which is one of the mechanisms that leads to the constant maintenance of glucose level (homeostasis) in the human body (Figure 2D).

Aside from extracellular rhythms involved in insulin secretion, such as melatonin rhythms, isolated β-cells show cell-autonomous rhythms by maintaining and regulating the rhythmic insulin secretion independently (Pulimeno et al., 2013; Perelis et al., 2015). This endogenous rhythmicity is known as basal insulin secretion (Saini et al., 2016). Basal insulin secretion is only between 0.5 and 1.0 units per hour and seems insignificant compared to the total amount of about 40 units of insulin secreted in an adult in a day (Ramchandani et al., 2010). However, basal secretion occurs continuously during the fasting periods throughout the day and accounts for approximately 50% of the total daily insulin secretion (Ramchandani et al., 2010). In *ex vivo* and *in vitro* models of the pancreatic islets, master circadian clock genes called *CLOCK* and *ARNTL* regulate synchronous cell-autonomous rhythms (Perelis et al., 2015; Saini et al., 2016). These clock genes regulate intracellular Ca^2+^ signaling pathways; the disruption of normal functions of *ARNTL* results in the inhibition of intracellular Ca^2+^ rhythms (Cavieres-Lepe and Ewer, 2021). The Ca^2+^ rhythm is an essential factor in insulin secretion and at the level of individual β-cells, receptor-mediated glucose uptake generates increased levels of adenosine triphosphate (ATP), leading to membrane depolarization followed by the opening of Ca^2+^ channels. Consequently, this influx of extracellular Ca^2+^ into β-cells activates the insulin secretory machinery and release of insulin from the cells (Campbell and Newgard, 2021). Synchronized rhythmic insulin secretion from a group of β-cells within an islet is mediated by Ca^2+^ flux through the gap junction channels that connect adjacent β-cells (Daraio et al., 2017; Idevall-Hagren and Tengholm, 2020). Accordingly, there is a possible interaction between the clock genes (central rhythm), Ca^2+^ signaling (internal rhythm), and synchronous insulin secretion (consequential rhythm) that contributes to the continuous regulation of glucose levels (homeostasis; Figure 2E).

## Biological rhythms in the context of mechanical stimulation of cell cultures

Given the above, biological rhythms are regulated both endogenously and exogenously, which occur cooperatively to regulate complex biological processes and maintain homeostasis in the system. We have classified these rhythms as a trio involving central rhythms, internal/external rhythms, and reflex/consequential rhythms, and the connections between the rhythms are essential. We will now discuss the relevance of biological rhythms in the context of mechanical stimulation of cell cultures.

When mechanically stimulating cell cultures for tissue engineering and regenerative medicine, it is necessary to consider mechanical parameters, which take place in the position of external rhythms in Figure 2A, as rhythmic variables and not as “static” variables. Rhythmic mechanical stimulation of cells could be organized as micro-rhythms (milliseconds, seconds, and minutes; ultradian rhythms) and macro-rhythms (~24 h and days; circadian and infradian rhythms). Here, we discuss the importance of this consideration in relation to the fact that the cellular mechanical response and sensitivity, which take place in the position of reflex/consequential rhythms in Figure 2A, are rhythmic (Thompson et al., 2020), rapid (ion channel activation; Matthews et al., 2010), and reduced over time (aging; Yang et al., 2017).

Firstly, cellular mechanical sensitivity and response can be rhythmic. It has been shown previously that cellular clock genes can regulate mechanical cellular functions such as cell migration—directed cell movement or change of position—in fibroblasts (Hoyle et al., 2017), resulting in showing patterns in cell migration over time. One of the cellular mechanisms that is actively involved in cell migration and mechanosensing is the cytoskeleton—a cellular component that is mainly responsible for the mechanical and structural aspects of the cells (Dominguez and Holmes, 2011). The dynamic structural alterations of F-actin filaments—a subcomponent of the cytoskeleton—in the form of lamellipodia and filopodia drive the migration at the cell front (Krause and Gautreau, 2014). Interestingly, the dynamics of F-actin filament formation can also be rhythmic. This is evident through the rhythmic intracellular expression of cofilin (Hoyle et al., 2017), a protein that regulates actin dynamics (Bravo-Cordero et al., 2013). Another example of rhythmic activities of the cytoskeleton is the fluctuating rate of wound healing which exhibits circadian rhythms where wounds (fibroblast cell cultures, skin wounds in rodents, and burn injuries in humans) are healed faster during the daytime (Hoyle et al., 2017). Furthermore, Ihara et al. (2017) illustrated that clock genes, such as *CLOCK* and *ARNTL*, can regulate the mechanosensing of the mucosa in the bladder of rodents. Healthy rodent models showed rhythmic expression of the mechanosensors, *Connexin26* (*Cx26*) and vesicular nucleotide transporter (*Vnut*), in the mucosa, which is more active during the day than at night. The disruption of the clock genes resulted in disturbed rhythmicity of the mechanosensing of *Cx26* and *Vnut* and showed an abnormally sensitive bladder during the night (Ihara et al., 2017).

Secondly, cellular sensitivity and response to mechanical stimulation can be rapid. In the process of fast cellular mechanosensing, integrins—transmembrane proteins that mediate the adhesion of cells to the extracellular matrix—play a central role (Chen et al., 2017; Martino et al., 2018). Integrins are also essential components of the focal adhesion (FA) points—multiprotein complexes that link the extracellular matrix to the actin filaments of the cytoskeleton (Wu, 2007). Generally, mechanotransduction—intracellular conversion of sensed mechanical stimulus into electrochemical signals—of integrins occurs within 500 ms after the cells were mechanically stimulated (Strohmeyer et al., 2017). However, it has been shown that the initiation of integrin-mediated intracellular Ca^2+^ influx happens as prompt as four milliseconds after mechanical stimulation was applied directly to the integrins, although the Ca^2+^ influx only peaked around 300 to 400 ms after the mechanical stimulation (Matthews et al., 2010). Moreover, integrin-mediated activation of the FA protein *SRC*—a signaling protein involved in cellular processes like migration, division, and differentiation—has been shown to take around 300 ms (Na et al., 2008). These dynamic and fast-responding cellular mechanisms are closely interrelated with cellular rhythms. For instance, *NR1D1*—a circadian rhythm clock gene—regulates FA formations in fibroblast cultures (Cunningham et al., 2020). Additionally, changes in the mechanical stiffness of the microenvironment are sensed by FA complexes and can lead to both altered circadian rhythmicity in mammary and lung epithelial cell cultures (Yang et al., 2017) and changes in rhythmic Ca^2+^ signaling between smooth muscle cells (Stasiak et al., 2020).

Thirdly, rhythmicity in cells and tissues dampens with age, which has been suggested to be partially due to the stiffening of tissue (Yang et al., 2017). The stiffening of *in vivo* tissue has a significant impact on the homeostasis of the human body in general (Sherratt, 2013; Heinz, 2021; Ryu et al., 2021). By using *in vitro* models, the substrate or extracellular matrix stiffness can be altered to mimic the physiological changes observed during aging to illustrate reduced rhythmicity. Accordingly, mammary and lung epithelial cells grown in a soft microenvironment (3D culture with stiffness ~30 Pa) exhibited functional circadian rhythmicity, whereas cell cultures grown in a hard microenvironment (2D culture with stiffness >100 MPa) exhibited reduced rhythmicity (Yang et al., 2017).

Collectively, there is adequate evidence to show that cellular processes of mechanotransduction are rhythmic, and these rhythmic cellular processes may occur in different temporal scales (micro- and macro-rhythms). A deeper understanding of mechanotransduction is crucial since many diseases arise from cellular mechanotransduction defection (Jaalouk and Lammerding, 2009). The effects of micro- and macro-rhythmic mechanical stimulations have been reported in recent experimental findings. For instance, Rogers et al. reported on the effect of rhythmic mechanical stimulation using a flexible silicone growth substrate stretching at regular rhythmic intervals (the frequency of 1 Hz) on human stem cell cultures. Following cycles of 12 h of regular rhythmic stretching and 12 h resting period for three days, they demonstrated synchronization of the clock genes (*ARNTL*, *PER1*, *PER2*, and *NR1D1*) in human stem cells (Rogers et al., 2017). Moreover, Vágó et al. demonstrated the effect of rhythmic mechanical stimulation using a uniaxial compression force on chondroprogenitor cells (from chicken). The stimulation was one h/day for six days and rhythmic mechanical stimulation entrained circadian clock genes (*ARNTL*, *CRY1*, and *PER3*), leading to enhanced tissue homeostasis and histogenesis (Vágó et al., 2021). In both studies, the trio rhythm model has been demonstrated: synchronization of specific clock genes (central rhythm), rhythmic mechanical stimulation (external rhythm), and cellular responses (e.g., stem cell differentiation capability and tissue homeostasis; consequential rhythms).

The regulation of clock genes without chemical or temperature-related stimuli potentially increases the utility of tissue engineering research in terms of cell transplantation, apart from personalized medicine and drug screening, even further. In particular, a clock gene such as *ARNTL* is reported to be an important factor in the WNT signaling pathway (Guo et al., 2012), which is a crucial stem cell mechanism that initiates the differentiation process—a stem cell process when its potential is lost and forms into adult cells, for example, cardiac muscle cells or skin cells. These findings are encouraging, but it must be noted that stem cells from different locations in the body respond differently to the same type of rhythmic mechanical stimulation (Rogers et al., 2017). Therefore, more extensive studies on optimization of the rhythmic mechanical stimuli that can closely mimic *in vivo* cellular environments for cell cultures are necessary.

In particular, microphysiological systems or organ-on-chips provide a great advantage in growing and studying cellular responses in dynamic cell culture systems (Wikswo, 2014). Compared to conventional static cell culture systems, implementing cell cultures in microfluidic chip systems offers the possibility to mimic key aspects of human physiology more accurately, including rhythmic stimulation (Zhang et al., 2018). Thus, growing cells in micro-devices allow to control (magnitude and rhythmicity), mechanical forces (e.g., stretching, pulling, compression, and shear forces), chemical signaling (e.g., growth factors, hormones, and nutrients), and electrical stimulation that the cells are exposed to (Ergir et al., 2018).

## Conclusion

In this article, we have classified biological rhythms using a trio model (central rhythms, internal/external rhythms, and reflex/consequential rhythms). It is imperative that all three rhythms in the trio function continuously to regulate homeostasis in a given biological system. Thus, the link between the three rhythms is important and relevant in tissue engineering and regenerative medicine as rhythmic interactions, whether in micro- or macro-rhythms, are vital in the early development of endogenous biological rhythms. This is evident through the lack of endogenous rhythms (e.g., transcriptional-translational feedback loop) in embryonic stem cells in general compared to adult stem cells (e.g., bone marrow mesenchymal stem cells; Rogers et al., 2017). For instance, circadian rhythmic patterns observed in neonates’ heart rates disappear shortly after birth and return only 3 to 4 weeks later (Ardura et al., 1997). The rhythmicity observed in the early neonatal stage is presumed to be due to maternal influence and endogenous rhythmicity is fully developed only at a later stage, but there are conflicting views on whether external rhythms (e.g., light and dark cycles) have any effect on the development of circadian rhythms in neonates (Begum et al., 2006). Moreover, it has been shown previously that insulin secretion by pancreatic β-cells depends on external rhythms (e.g., fasting and feeding cycles) to develop endogenous rhythms and to be fully matured (Seshadri and Doucette, 2021). As these experimental observations illustrate, the importance of interactions between the rhythms in the entrainment and development of fully functioning biological rhythms should not be minimized.

As the name suggests, the trio rhythms are like a musical ensemble in that each rhythm is individually important, but they attentively listen and interact with one another and even with the audience to achieve a successful performance. Furthermore, as the ensemble starts to interact with other ensembles, it becomes a structure like an orchestral ensemble where the interactions between different sections of the orchestra are extremely sensitive and intricate to form some kind of homeostasis usually led by a conductor. The model is intentionally reductive and can be made more specific by exploring additional physical examples such as the seven-step model (stimulus, receptor, input, integrating center, output, effector, and response) presented by Chirillo et al. (2021). Still, we think it is also essential to try to pin down the core patterns from complex processes to get an overview and understanding of the discussed rhythms in this article at a macroscale. We hope that the trio model provides a framework that makes it possible to focus in and out of different spatial and temporal scales to get a basic but fundamental understanding of how biological, particularly cellular, rhythms function and interact with one another.

## Author contributions

DK, AD, and AJ contributed to the conception of the study. DK wrote the first draft of the manuscript. All authors contributed to the article and approved the submitted version.

## Funding

This work was supported by UiO:Life Science through the ABINO project and the Research Council of Norway through its Centres of Excellence funding scheme, project numbers 262613 (HTH) and 262762 (RITMO). The funders had no role in study design, data collection, and analysis, decision to publish or preparation of the manuscript.

## Acknowledgments

We would like to thank Hanne Scholz (Hybrid Technology Hub, University of Oslo) and Finn Upham (RITMO, University of Oslo) for their valuable comments on the manuscript.

## Conflict of interest

The authors declare that the research was conducted in the absence of any commercial or financial relationships that could be construed as a potential conflict of interest.

## Publisher’s note

All claims expressed in this article are solely those of the authors and do not necessarily represent those of their affiliated organizations, or those of the publisher, the editors and the reviewers. Any product that may be evaluated in this article, or claim that may be made by its manufacturer, is not guaranteed or endorsed by the publisher.
